# *NEFL* is overexpressed and it modulates invasion and migration in neuroendocrine-like PC3-ML2 prostate cancer cells

**DOI:** 10.17912/micropub.biology.000658

**Published:** 2022-10-22

**Authors:** Tanya C Burch, Stephen Mackay, Naomi L Hitefield, Autumn B Roberts, Ian O Oduor, Julius O Nyalwidhe

**Affiliations:** 1 Eastern Virginia Medical School; 2 Microbiology and Molecular Cell Biology; 3 Leroy T. Canoles Jr. Cancer Research Center; 4 Leroy T. Canoles Jr. Cancer Research Center

## Abstract

Prostate cancer clinical outcomes are varied, from non-aggressive asymptomatic to lethal aggressive neuroendocrine forms which represent a critical challenge in the management of the disease. The neurofilament light ( *NEFL* ) is proposed to be a tumor suppressor gene. Studies have shown that expression of the gene is decreased in various cancers. We have used quantitative RT-PCR, immunoblotting, methylation specific PCR, siRNA knockdown followed by migration/invasion assays to determine associations between *NEFL* expression and disease phenotype in a panel of prostate cells. We demonstrate that *NEFL* is overexpressed and it modulates invasion and migration in PC3-ML2 prostate cancers cells which have an aggressive neuroendocrine-like phenotype.

**Figure 1. NEFL is overexpressed in prostate cancer cell lines with neuroendocrine like phenotypes and knockdown cells have reduced migration and invasion capacity.**
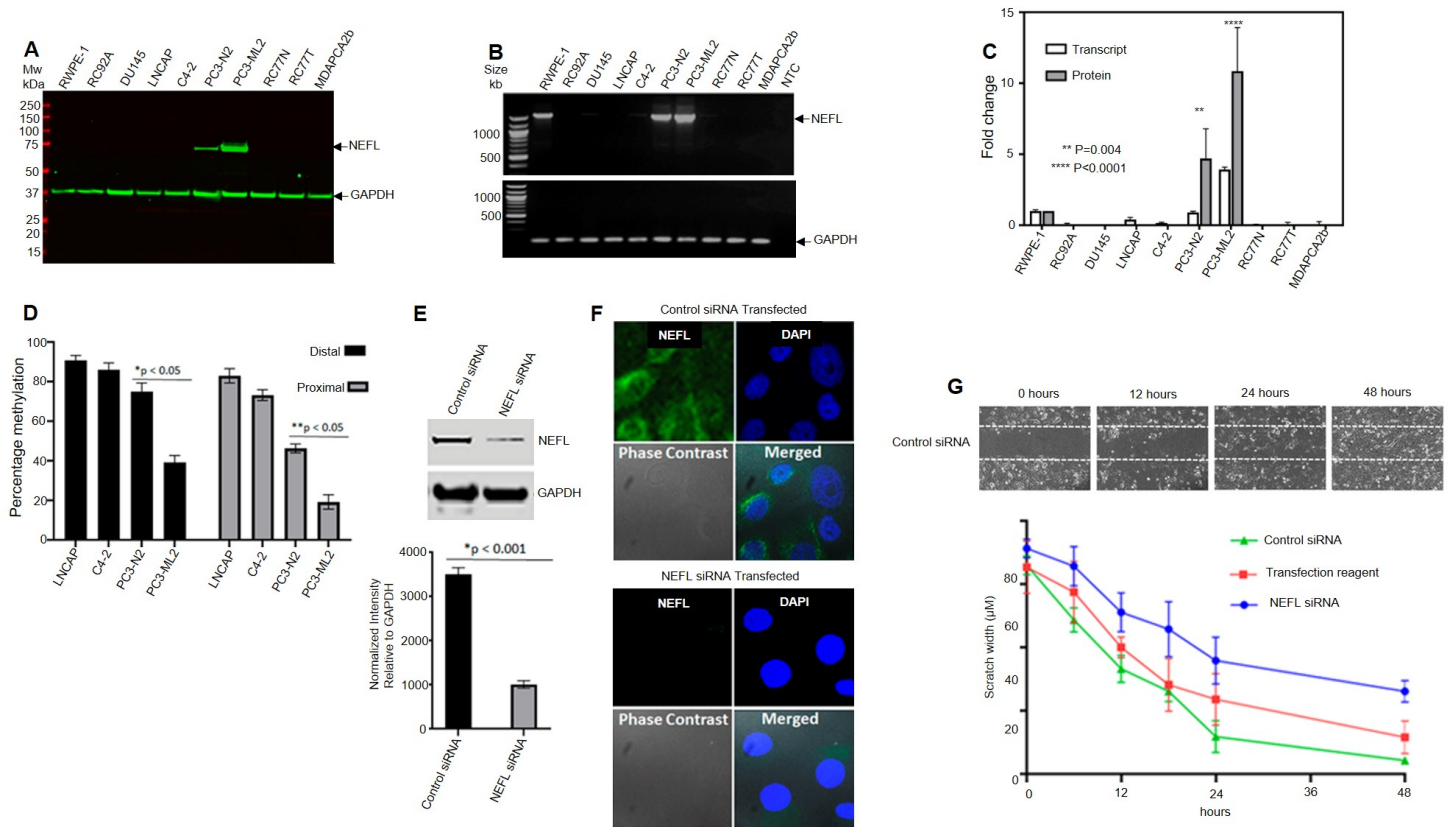
**(A)** Semi-quantitative mRNA expression of *NEFL* in 10 prostate cell lines. cDNA was synthesized from total mRNA extracted from 10 prostate cell lines and used for RT-PCR to evaluate the expression of *NEFL* in the cells. GAPDH was used as a control for normalization. **(B)** Western blot detection of NEFL protein in 10 prostate cancer cell line and GAPDH was used as a loading control. **(C)** Expression fold change of NEFL protein and transcript are based on normalization by individual GAPDH values. There is a significant enhanced expression of NEFL protein and transcript in PC3-N2 and PC3-ML2 cells compared to control non cancer RWPE-1 cells. **(D)** The methylation status of four prostate cancer lines was assayed by MSP for the *NEFL* distal (1319-1400) and proximal (1957-2047) promoter regions. RT-PCR amplification was performed using primers specific to these regions, and beta-actin was used as an internal control. QPCR Ct values were used to calculate percentage of CpG methylation of the target regions. There is a significant reduction in *NEFL* methylation in the neuroendocrine-like PC3 cells. **(E)** Western blot detection of *NEFL* after siRNA transfection of PC3-ML2 cells with *NEFL* targeting nucleotides and control scrambled nucleotide sequences for 72 hours. **(F)** Immunofluorescence confocal microscopy detection of PC3-ML2 transfected with scrambled control siRNA and *NEFL* targeting siRNA. **(G)** NEFL silencing significantly reduces the migration and invasion ability of PC3-ML2 cells. The migration and invasive capacity of PC3-ML2 cells was evaluated in scratch/wound healing assays using non-transfected control, control siRNA and *NEFL* siRNA transfected PC3-ML2 cells. Migration towards the wound area and scratch was measured after every 6 hours to maximum of 48 hours in triplicate experiments. The migration rates between the non-transfected control and control siRNA versus *NEFL* siRNA transfected cells are significantly different in triplicate experiments. The *NEFL* siRNA transfected cells have a lower migratory potential compared to the controls (p<0.05). Images of control siRNA transfection experiments at different time intervals and the quantitation graph encompassing data from the three experiments in performed in triplicate are shown.

## Description

Prostate cancer (PCa) is the most common cancer in men in USA. For the year 2022, current estimates predict the diagnosis of 268,490 new cases and 34,500 deaths in the country (Siegel et al., 2022) Prostate cancer has diverse disease pathogenesis and clinical outcomes. Some patients develop insignificant indolent disease whereas others develop highly aggressive forms of the disease with lethal consequences. PCa pathogenesis is associated with the progression to a neuroendocrine phenotype at the late stages (Aggarwal and Small, 2014; Kanayama and Luo, 2021). This is usually lethal and most patients die in less than one year. The mechanisms that mediate aggressive disease progression have not been completely characterized and or understood. Several factors including tumor suppressors and oncogenes play a central role in malignant transformation and progression to aggressive disease. The presence of inactive tumor suppressor genes (TSGs) often characterize aggressive cancers.

Neurofilaments proteins (NF) are type IV intermediate filament heteropolymers composed of light, medium, and heavy chains. These proteins are constituents of the axoskeleton and they functionally maintain the neuronal caliber. The neurofilament light (gene name *NEFL* Synonyms: NF68, NFL), has been proposed to be a tumor suppressor gene in different cancers. *NEFL* is located at chromosome 8p21 loci where mutations heterozygous and homozygous and loss of heterozygosity are observed in several cancers (Macoska et al., 1995; Vocke et al., 1996; Häggman et al., 1997; Takimoto et al., 2001; Coon et al., 2004; Burke et al., 2006; Schmidt et al., 2007). Methylation which results in the silencing and inactivation of TSGs has been reported for *NEFL* in different cancers, and this may play a role in cancer progression and aggressive disease (Calmon et al., 2015; Dubrowinskaja et al., 2014; Kang et al., 2013; Revill et al., 2013; Li et al., 2005). Neurofilament proteins are mostly abundant in most abundant in the nervous system. Functionally, NEFL protein maintains neuronal caliber and is involved in the transportation of neurotransmitters to axons and dendrites (Herrmann et al., 2009; Eriksson et al., 2009; Liem et al., 2009; Steinert et al., 1988; Friede et al., 1970). The role of *NEFL* gene in prostate cancer initiation and progression is still incompletely understood. Published data demonstrate that neurofilament genes are differentially expressed in multiple cancers and that the *NEFL* gene likely plays a role in cancer development and progression (Calmon et al., 2015; Dubrowinskaja et al., 2014; Kang et al., 2013; Li et al., 2012; Huang et al., 2014; Shen et al., 2016; Capasso et al., 2014; Chen et al., 2021; Fan et al., 2022). To further understand the role of *NEFL* in prostate cancer pathogenesis, we have determined the expression profile of NEFL gene in a panel of 10 cell lines that include a normal epithelial prostate cancer cell line and nine prostate cancer cell lines with different malignancy phenotypes. The origin and other characteristics of the cells are provided in the **Reagents Table** . We have used RT-PCR, Western blot, and methylation specific PCR (MSP) to determine the expression profile of *NEFL* in a panel of 10 prostate cell lines.

Our findings demonstrate a unique increased expression of *NEFL* in the highly metastatic androgen independent PC3 cell derivatives, which have a neuroendocrine-like phenotype **(Figures 1A-C)** . These two syngeneic cell lines PC3N2 and PC3-ML2 have different tumorigenic phenotypes. Unlike PC3-ML2 cells which are highly tumorigenic in vitro and with skeletal bone metastases in vivo, the N2 cells do not migrate through matrigel-coated membrane in vitro and they do not induce skeletal metastases in SCID mice. *NEFL* expression is significantly upregulated (>3 fold) in the PC3-ML2 versus the non-metastatic PC3-N2 cells. Although there is an mRNA signal for the NEFL transcript for RWPE-1 cells which is a normal epithelial prostate cancer cell line (Figure 1A), the protein is translated and expressed at extremely low levels (Figure 1C), and with >5 fold upregulation in PC3-ML2 versus RWPE1-1. *NEFL* is not expressed in the other cell lines which include the androgen dependent LNCAP and its derivative C4-2 cell lines. In LNCAP and C4-2 cells, which have a common origin and background, the more aggressive C4-2 cells exhibit lower methylation as compared to LNCAP cells. In syngeneic PC3-N2 and PC3-ML2 cells, the methylation status of these regions decreases with increased aggressiveness with the lowest levels of methylation observed in the aggressive PC3-ML2 which have higher invasive capacity and metastatic potential amongst the two cell lines **(Figure 1D)** . In addition, siRNA mediated *NEFL* knockdown inhibits the migration capacity of PC3-ML2 Cells in a scratch/wound healing assay (Figures 1E-G). There are slight differences in the wound healing assay result between control siRNA transfection and Transfection reagent but both are significantly different from the NEFL siRNA transfection experiment.

Overall, we show that NEFL expression occurs in a subset of prostate cancer cells with neuroendocrine-like phenotype that is associated with aggressive prostate cancer and siRNA silencing of NEFL inhibits the migration and invasion potential of PC3-ML2 cells.

## Methods

Cell Lines and Culture Conditions : A panel of one normal epithelial prostate cell line and nine prostate cancer cell lines were used in the study. Four cell lines RWPE-1, LNCAP, C4-2, DU-145 and MDAPCa2b were directly obtained from the American Type Culture Collection (ATCC). Three cell lines RC92A, RC77N-E and RC77T-E were obtained from Dr. Johng S. Rhim (USUHS) and they have been described previously (Theodore et al., 2010). Two cell lines PC3-N2 and PC3-ML2 were obtained from Dr. Stearn and their development has been described (Wang et al., 1991). The characteristics of 10 cell lines are shown and summarized in reagents.

RT-PCR Analysis and Western Blot Analysis: Total RNA was extracted from the 10 prostate cell lines using (RNeasy; Qiagen), and used for cDNA synthesis using SuperScript II reverse transcriptase kit according to manufacturer’s instructions (Invitrogen). *NEFL* and *GAPDH* specific transcripts were targeted using primers designed to generate PCR products with approximately the same number of base pairs. GAPDH was used as a housekeeping gene control for normalization. The primers for *NEFL* were as follows: forward, 5′-ATGAGTTCCTTCAGCTACGA -3′ and reverse, 5′-TCAATCTTTCTTCTTAGCTGC-3′, and GAPDH forward, 5′-CAGCCTCAAGATCATCAGCA-3′ and reverse, 5′-ACAGTCTTCTGGGTGGCAGT-3’. The *NEFL* and *GAPDH* expression profiles as determined by obtained RT-PCR were further validated in the 10 cell lines using immunoblots as we have previously described (Burch et al., 2013; Burch et al 2015). The anti-NEFL antibody (sc-20012, Santa Cruz Biotechnology) was used to determine the expression levels of the protein in the 10 cell lines. GAPDH (sc-25778, Santa Cruz Biotechnology) was used as the protein loading control.

Promoter Region Methylation Analysis: DNA was isolated based on the protocol for kit P-1018, FitAmp Blood and Cultured Cell DNA Extraction Kit (Epigentek, Inc.). DNA was eluted in TE buffer in a total volume of 30 µl and the DNA was quantified by Nanodrop spectroscopy. Bisulfite modification was performed using 100 ng of purified DNA based on the protocol for the P-1001 Methylamp DNA Modification Kit (Epigentek, Inc.). Methylated HeLa CpG DNA was included as a control. QPCR was performed in duplicate using 1 µl of modified DNA and gene-specific primers designed for modified DNA template. For the *NEFL* promoter region (Acc# L04147.1), two genomic regions were targeted: Distal region (1319-1400) covered by primer pairs un-methylated 1 (UM1) or methylated 1 (M1) and Proximal region (1957-2047) covered by primer pairs un-methylated 2 (UM2) or methylated 2 (M2). The primer sequences are as follows: UM1 forward 5'-GTTAAAGTTATTTGTGGTTT-3’, reverse 5’-TACAACAATTAATCCACCT-3'; M1 forward 5'-ACGTTAAAGTTATTTGCGGT-3', reverse 5'-TTACAACAATTAATCCGCCT-3'; UM2 forward 5'-TTTGGGTTGTAGTTTGATTT-3', reverse 5'-CACAACACACCAACCAATAA-3'; M2 forward 5'-TTCGGGTCGTAGTTCGATTT-3', reverse 5'-ACGACACGC CGACCAATAA-3'. Real time quantitative PCR amplification was performed for 40 cycles of 98 °C for 30 s, 55 °C for 30 s, and 72 °C for 30 s, with 5 min at 95 °C for initial denaturation and 5 min at 72 °C for final elongation as described by Shen et al 2016. Beta-actin was used as an internal control to determine bisulfite conversion efficiency. Data analysis was performed using QPCR Ct values to calculate CpG methylation % of each target region using the following expression: % methylation = {1-[2(Ct M- Ct UM) / (2(Ct M- Ct UM) +1)]} x 100%.

Transfection and Silencing of *NEFL* by siRNA: The expression of the *NEFL* gene in PC3-ML2 cells was silenced using small interference RNA specific to the gene (sc-36048, Santa Cruz Biotechnology Inc.). Scrambled non-targeting siRNA (control siRNA-A, sc-37007, Santa Cruz Biotechnology Inc.) were used in negative control experiments. The control siRNA consists of a scrambled 20-25 non-targeting sequence negative control that do not lead to the specific degradation of any cellular message. The transfections were performed using 20 nM of the siRNA duplexes according to the manufacturer’s protocol from Santa Cruz Biotechnology Inc. (Santa Cruz, CA). The transfection reactions with *NEFL* and control siRNA were done on six well plates and allowed to proceed for 72 hours before further biochemical and imaging analyses. The cells were harvested and processed for Western blot analysis and for immunofluorescence detection of NEFL by confocal microscopy to validate efficient silencing of *NEFL* gene expression upon siRNA transfection. Another batch of transfected cells were used in scratch/wound healing assays to determine the effects of the knockdown on the migration and invasive properties as we have previously described (Burch et al., 2013).

Confocal Microscopy Analysis: NEFL protein expression in PC3-ML2 cells was determined using confocal microscopy as we have previously described (Burch et al., 2013).

Scratch-Wound Healing Invasion Assay: The effects of *NEFL* silencing on the migration and invasion capacity of PC3-ML2 cells was determined using a scratch/wound healing assay as previously described (Burch et al., 2013) Transfections were performed in triplicate experiments using non-targeting and *NEFL* specific siRNA as described in the preceding section using 2 x 10 ^5^ cells per well. To determine the rates of invasion, images of the scratch were taken immediately after the scratch, at 0 hours, and then after every 6 hours to a maximum of 48 hours. The migration distances in the wounds were measured, and the mean and standard deviation values in the triplicate experiments were calculated and the differences compared between the control-non-transfected, scrambled control siRNA transfected and *NEFL* siRNA transfected PC3-ML2 cells.

Statistical Analysis: All the experiments were performed in triplicate. Differences between experimental variables of the groups was determined using two two-tailed Student t-tests to determine differences with a p-value threshold of < 0.05 being significant. Statistical analyses were performed using SPSS (SPSS Incorporated, Chicago, USA).

## Reagents

**Table:** 

**Cell Line**	**Origin**	**Characteristics**
RWPE-1	Immortalized (HPV) prostate epithelial cell line from a normal 54 year old Caucasian male	Non-malignant, non-metastatic, does not form tumors, androgen sensitive, expresses PSA
RC92A	Telomerase-immortalized malignant cells from a 57 year old Caucasian American male	Primary adenocarcinoma
DU145	Adenocarcinoma brain metastatic tumor from a 69 year old Caucasian male	Moderately metastatic, not hormone sensitive, does not express PSA
LNCAP	Adenocarcinoma supraclavicular metastatic lymph node tumor from a 50 year old Caucasian male	Low metastatic potential develops tumors in mice, hormone sensitive, expresses PSA
C4-2	LNCAP subline	Tumorigenic and metastatic, androgen insensitive, lower PSA expression, reduced AR expression
PC3-N2	PC3 subline. PC3 is an Adenocarcinoma bone metastatic tumor from a 62 year old male Caucasian	Less aggressive compared to PC3-ML2, hormone refractory, does not form metastases when injected in SCID mice
PC3-ML2	PC3 subline. PC3 is an Adenocarcinoma bone metastatic tumor from a 62 year old male Caucasian	More aggressive compared to PC3-N2, hormone refractory, form metastases when injected in SCID mice
RC77N/E	Immortalized (HPV) non-malignant cells from a normal 62 year old African American male	Non-malignant
RC77T/E	Immortalized (HPV) prostate adenocarcinoma cells from a normal 62 year old African American male	Primary adenocarcinoma
MDAPCa2b	Adenocarcinoma metastatic bone tumor from a 63 year old African American male	Adenocarcinoma metastatic bone tumor, hormone refractory
